# Spatio-Temporal Variation of Longevity Clusters and the Influence of Social Development Level on Lifespan in a Chinese Longevous Area (1982–2010)

**DOI:** 10.3390/ijerph14070812

**Published:** 2017-07-19

**Authors:** Jian Qin, Guoqi Yu, Tianlong Xia, You Li, Xue Liang, Peng Wei, Bingshuang Long, Mingzhi Lei, Xiao Wei, Xianyan Tang, Zhiyong Zhang

**Affiliations:** 1 Department of Environmental and Occupational Health, Guangxi Medical University, Nanning 530021, China; qinjian@gxmu.edu.cn (J.Q.); qiguoyuer@163.com (G.Y.); xiatianlong@126.com (T.X.); liyou121300@163.com (Y.L.); 490275305@139.com (X.L.); gxwp2016@126.com (P.W.); 15677188715@163.com (B.L.); m18677111761@163.com (M.L.); gxmu_xwei@126.com (X.W.); 2 Department of Epidemiology and Health Statistics, Guangxi Medical University, Nanning 530021, China

**Keywords:** longevity, spatio-temporal variation, centenarians, social development, gravity modeling

## Abstract

The study aims to determine the spatial and temporal variation of a longevous region and explore the correlation between longevity and socioeconomic development. Population data at the township level were obtained from the last four population censuses (1982–2010). Five main lifespan indicators and the Human Development Index (HDI) were calculated. Getis-Ord G*, Gravity modeling, and Pearson’s *r* between lifespan indicators and HDI were applied. In this study, a stable longevous gathering area was discovered in Hechi during different periods. Under the influence of social and economic development, more longevous areas appeared. However, the effects of genetic and natural environmental factors on longevity were always dominant in this remote and mountainous city. Furthermore, longevity indicators lacked any significant correlation with life expectancy. No significant positive correlation was detected between lifespan indicators and HDI. Thus, we conclude that lifespan indicators can determine the spatial distribution and variation pattern of longevity from multiple dimensions. The geographical scope of longevity in Hechi City is gradually expanding, and significant spatial clustering was detected in southwestern, southern, and eastern parts of Hechi. This study also found that social economic development is likely to have a certain impact on new longevous areas, but their role on extreme longevity is not significant.

## 1. Introduction

Health and longevity are crucial concerns in today’s world. The secret of longevity has been explored for thousands of years in China. Studies have shown that longevity is the result of a combination of multiple factors. Genetic, environmental and other unknown factors may influence the human life span to some extent [1,2]. Genetic effects accounted for only 20–30% of the formation of longevity, whereas environmental factors play a dominant role [3]. Thus far, several geographical longevous areas (including Sardinia, Nicoya, Ikaria and Bama) and some economic longevity areas (including Sweden and Japan) were identified [4,5]. The former are often located in poor mountainous areas or isolated islands, mainly affected by the physical and geographical environment, including geography, atmosphere, water quality and so on, whereas the latter are mainly located in developed countries which benefit more from good medical services, diet, and behavior habits. It is well known that good socioeconomic conditions are beneficial to people for getting more healthy resources, thus reducing disease and improving lifespan [6,7], but to what extent socio-economic development can promote longevity and help people achieve extreme longevity, we do not yet know [8]. Therefore, we hope that through the study of the longevity of the Bama, we can get more clues.

Bama Yao Autonomous County is a famous longevous zones around the world. Studies have shown that longevity in the Bama region is closely related to the local superior geographical environment and related genes [9,10,11]. However, the results of the sixth national census showed that the proportion of centenarians in other counties of Hechi City in Southern China is increasing, which also prompted Hechi to be rated as one of the world’s longevous cities. We wondered if there are other longevous areas in this region, apart from Bama, and the relationship between the emergence of new longevous areas and socio-economic development in the past three decades. Accordingly, we conducted a longitudinal and cross-sectional study on the longevity of the Hechi area based on data from the previous four censuses. To measure the level of longevity, the following variables were selected: the number of centenarians per one hundred thousand inhabitants (CH), the ratio between the population above 90 years of age and the total population above 65 years of age (LI), the percentage of the population aged at least 80 years (ultra-octogenarian index, UOI), the ratio of the population above 80 years of age over the elderly population above 60 years of age (ultra-octogenarian of the ultra-sexagenarian, UOOUS), and life expectancy at birth (LE). CH and LI are relatively common indicators to identify extreme longevity [12,13], whereas UOI and UOOUS represent the general level of longevous people can either reach or achieve in most countries in the world. These four indicators which were derived from the population age structure have high representation, availability and sensitivity, even though they are affected to some extent by birth rate, mortality, and population migration [14]. LE refers to the length of life that a virtual population can achieve in accordance with the number of deaths in a certain period of time. LE can eliminate the effect of the age structure of the population and can be compared between different countries and regions. First published in 1990, the Human Development Index (HDI) was intended to be a simple and transparent device to compare the progress in human development; specifically, HDI is an aggregate of life expectancy, education, and gross domestic product per capita (GDP_PC_) [15]. Compared with simple economic indicators, it is a representative indicator to assess the overall development of a region which can reflect the balance between economic growth and social development, and the rational allocation of resources [16]. Most importantly, it can also indicate the fairness of health to some extent. In view of the above strengths, HDI has been a common indicator to evaluate and compare the level of social-economic development between different countries and regions in today’s world, although some scholars have questioned the selection of its constituent parts and particularly the weighting system [17,18]. Therefore, spatial analysis was used to explore the spatial heterogeneity and temporal trends of the above indicators.

The major objectives of this paper are: (1) to demonstrate the characteristics of the temporal and spatial variation of longevity from multiple dimensions and determine the clustering area of longevity; and (2) to explore the relationship between lifespan and HDI. This work may be a basis for policy recommendations for decision-makers in developing health strategies to help people achieve health and longevity.

## 2. Data and Methods

### 2.1. Study Area and Data Sources

**Figure 1:**
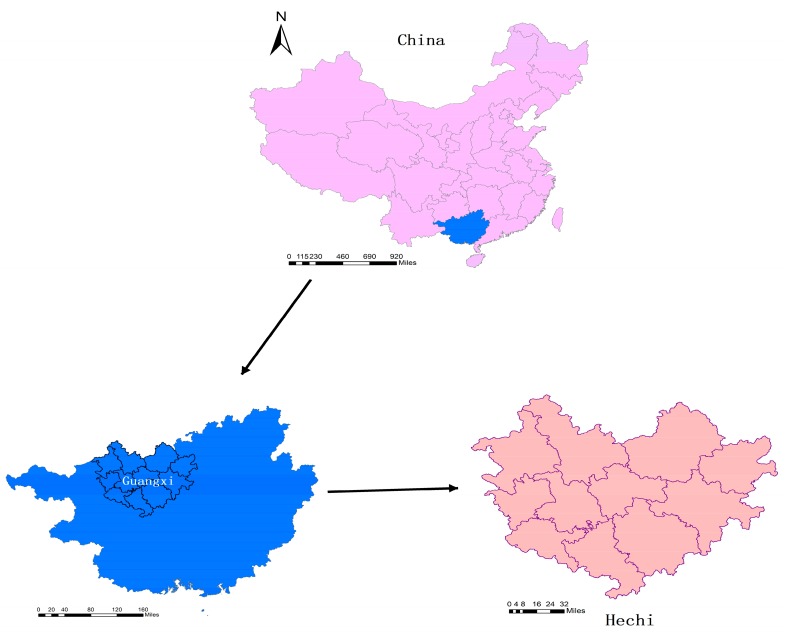
Location of the study area, Hechi city in China.

Hechi, located between latitude 23°41′ and 25°37′ north, and longitude 106°34′ and 109°09′ east, is a mountainous city in Southern China with a population of approximately 3.37 million. It comprises 139 townships belonging to 11 counties or districts with a total land area of 3350 km^2^, all of which are covered in the study (Figure 1).

We obtained the population data at the county- and township-levels from four national population censuses (1982, 1990, 2000, and 2010) [19,20,21,22]. Data on GDP_PC_ and part of the Educational status data were also collected [23,24,25,26]. Data on Dahua County in 1982 and Celing, Bachou Township in 1990 were not obtained. Hence, such figures were regarded as missing data from the various administrative regions during different time periods. Shapefiles used in this study to describe the distribution pattern of regional variation of lifespan indicators were vector files from the National Bureau of Surveying and Mapping. The map information was updated in 2011. The geographic coordinate system used was GCS_WGS_1984, while the corresponding projected coordinate system was the WGS_1984_World_Mercator.

### 2.2. Selection of Indexes

To better explore the characteristics of longevity, we chose the following statistical indicators: (1) CH, (2) LI, (3) UOI, (4) UOOUS, (5) LE –which was calculated according to the simple life table, and (6) (HDI, a composite index consisting of the life expectancy index, education index, and integrated gross enrollment index [27]. The above indicators are all widely accepted indicators to measure the level of longevity and economic development in China. These indicators were applied in a variety of studies on lifespan and extreme longevity.

### 2.3. Assessment of Reliability and Accuracy of Demographic Data

A complete, exhaustive, and detailed census could not be conducted for 30 years after the foundation of the People’s Republic of China because of economic and technical constraints. Therefore, results of the previous two censuses (1953 and 1964) were questioned by many domestic and foreign experts and scholars [28,29]. China had conducted a series of high-quality censuses in the following decades with the assistance of the United Nations Population Fund. The reliability and precision of the resulting data were guaranteed and are generally accepted [30]. The government has a set of rules to control the quality and authenticity of survey data, especially for age verification. Thus, we chose data from the last four censuses as the basis for the present study.

### 2.4. Statistical Analysis

We calculated the selected indicators using Excel 2016 (Microsoft, Redmond, WA, USA) for each population census. The method applied in the calculation of LE was given by Gompertz [31]. HDI is commissioned by the United Nations Organization to evaluate a country’s socioeconomic achievements in three basic aspects: longevity, knowledge, and standard of living. The specific algorithms of HDI are available from the website of the United Nations Organization [32]. We analyzed the correlation coefficients between the longevity indicators (CH, LI, UOI, and UOOUS) and LE, as well as the lifespan indicators and HDI, using SPSS 22.0 (IBM, New York, NY, USA). The distribution maps of the corresponding indicators, global autocorrelation analysis, curves of gravity center migration, and hotspot analysis were drawn with ArcGIS Geographic Information Systems software version 10.4.1 (ESRI, Redlands, CA, USA). The statistical significance involved in the study was set at *p* < 0.05.

#### 2.4.1. Spatial Autocorrelation Analysis

Global autocorrelation analysis was conducted in measuring the geographic patterns and clusters. Moran’s index (Moran’s *I*) was an important indicator of the analysis. Moran’s *I* can be expressed by the following formula [33]:

(1) $$I=\frac{n\sum_{i=1}^{n}\sum_{j=1}^{n}w_{ij}\left(x_{i}-\bar{x}\right)\left(x_{j-}\bar{x}\right)}{\left(\sum_{i=1}^{n}\sum_{j=1}^{n}w_{ij}\right)\sum_{i=1}^{n}{\left(x_{i}-\bar{x}\right)}^{2}}$$

In Equation (1), *n* is the number of spatial units indexed by *i* and *j*; *x* is the variable of interest; *x_i_* and *x_j_* are the values of the observed variable at sites *i* and *j*; $\bar{x}$ is the mean of *x*; and the weights *W_ij_* are written in a (*n* × *n*) weight matrix (the weight matrix depicts the relation between an element and its surrounding elements. In addition, weight can be based on contiguity relations or distance). The values of Moran’s *I* generally range from −1 to +1. Negative (positive) values indicate negative (positive) spatial autocorrelation. Values of Moran’s *I* can be tested based on their *Z*-scores and *p*-value which can indicate whether the null hypothesis is rejected or not. In this study, the null hypothesis states that the values of longevity indicators associated with geographic features are randomly distributed.

#### 2.4.2. Getis-Ord G*i Hotspot Analysis

Global autocorrelation can be used to analyze whether the attributes specified in the entire study area are relevant but it cannot exactly determine where such attributes are gathered. Thus, we use G statistics to reveal the spatial distribution pattern and the approximate spatial aggregation range. The G statistic is calculated as follows [34]:

(2) $$G_{i}=\frac{\sum_{j=1}^{n}w_{ij}\left(d\right)x_{j}}{\sum_{j=1}^{n}x_{j}}\;j\neq i$$

In Equation (2), *G_i_*(*d*) measures the degree of correlation between the value of position *i* and the value at each position *j* in the range of distance *d*. $w_{ij}\left(d\right)$ represent the spatial adjacency weight matrix within distance *d*. *Z*-scores and *p*-values from the tool are measures of statistical significance that indicate whether the observed spatial clustering of high or low values is more pronounced than one would expect in a random distribution of the same values.

#### 2.4.3. Simple Linear Correlation

The correlation coefficient is a statistical measure that can be used to determine the degree of linear correlation between two variables. It ranges from −1 to 1. The closer the absolute value of the correlation coefficient is to 1, the stronger the correlation will be. According to the different data characteristics, we can employ Pearson correlation coefficient or Spearman’s rank correlation coefficient [35,36].

#### 2.4.4. Mean Center

The mean center is the average $x^{-}$ and $y^{-}$ coordinate of all the features in the study area. This value is useful for tracking the changes in the distribution or for comparing the distributions of different research objectives. The weighted mean center is given as follows [37]:

(3) $$\bar{X}w=\frac{\sum_{i=1}^{n}w_{i}x_{i}}{\sum_{i=1}^{n}w_{i}},\;\bar{Y}w=\frac{\sum_{i=1}^{n}w_{i}y_{i}}{\sum_{i=1}^{n}w_{i}}$$

where $w_{i}$ is weighted at feature *i* (*i* refers to the administrative region in this study); $x_{i}$ and $y_{i}$ are the coordinates for feature *i*; and *n* is equal to the total number of features.

## 3. Results

### 3.1. Spatial Distribution and Variation of CH, UOOUS, UOI, LI, and LE at the County-Level in Hechi, China (1982–2010)

**Figure 2:**
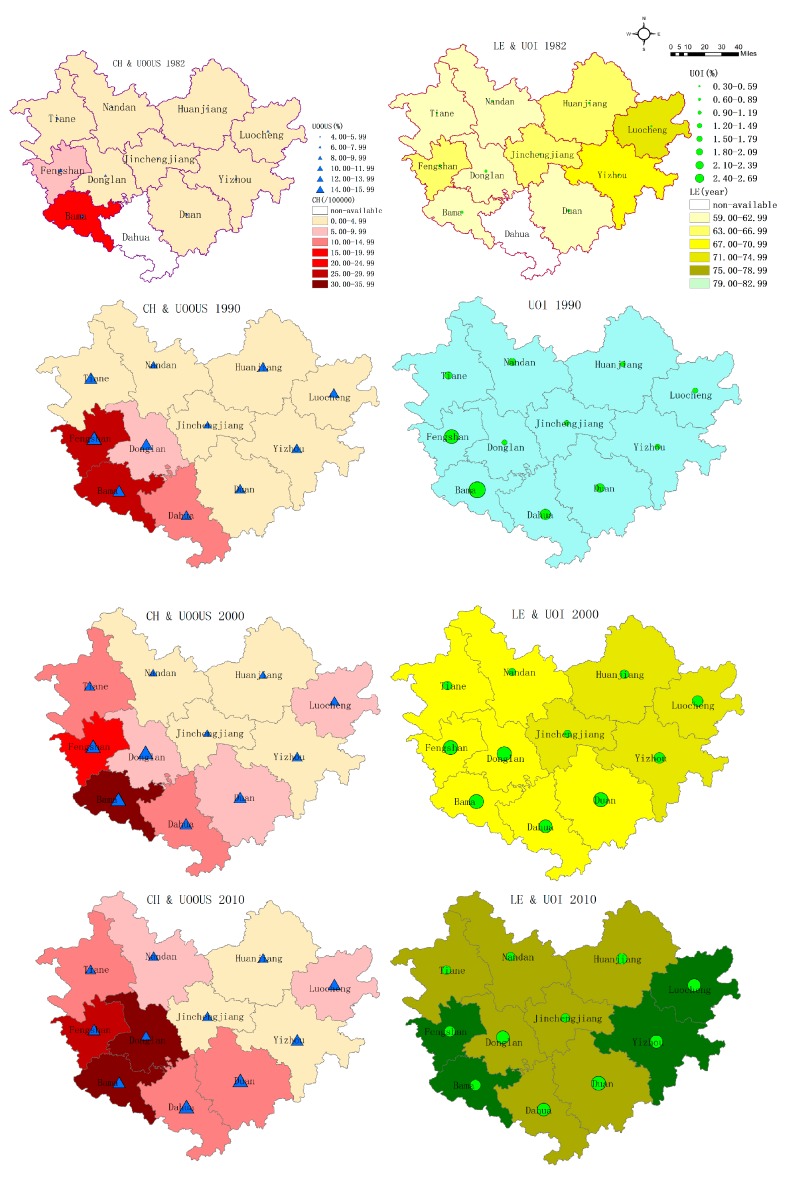
Spatial and temporal distribution of CH (the number of centenarians per one hundred thousand inhabitants), UOOUS (ultra-octogenarian of the ultra-sexagenarian; the ratio of the population above 80 years of age over the elderly population above 60 years of age), UOI (ultra-octogenarian index; the percentage of the population aged at least 80 years) and LE (the life expectancy at birth) in Hechi city at county level (1982–2010).

We calculated the lifespan indicators for four population censuses (1982, 1990, 2000, and 2010) and presented each of the indices in the form of a thematic map (Figure 2). Figure 2 indicates that Bama had the highest CH from 1982 to 2010. The high CH-value areas continued to expand from 1990. The CH value of Donglan grew rapidly and was close to that of Bama. Meanwhile, Fengshan maintained a high level of CH. A high-value area also gradually formed in the southwestern part of Hechi. In addition, LI exhibits almost the same pattern of variation as CH, whether from a vertical perspective of time or a horizontal comparison between study regions (as shown in Supplementary Materials Figure S1). The distribution and changes of UOOUS and UOI were consistent in the last four population censuses. Both were low in 1982, whereas a significant improvement was observed throughout the Hechi area by 1990. The value of the southwest region was initially high, while the eastern and southern regions showed substantial increase from 2000 and finally surpassed that of the southwest area. LE values at the county level were unavailable in 1990 because of the lack of statistical data. Based on the current LE data, the northeastern parts of Hechi exhibited more significant and stable differences compared with those of other areas. Bama and Fengshan also have high LE levels, especially in the recent decade, because of low mortality and the improvement of living standards brought about by tourism development.

### 3.2. Spatial Cluster Analysis

**Figure 3:**
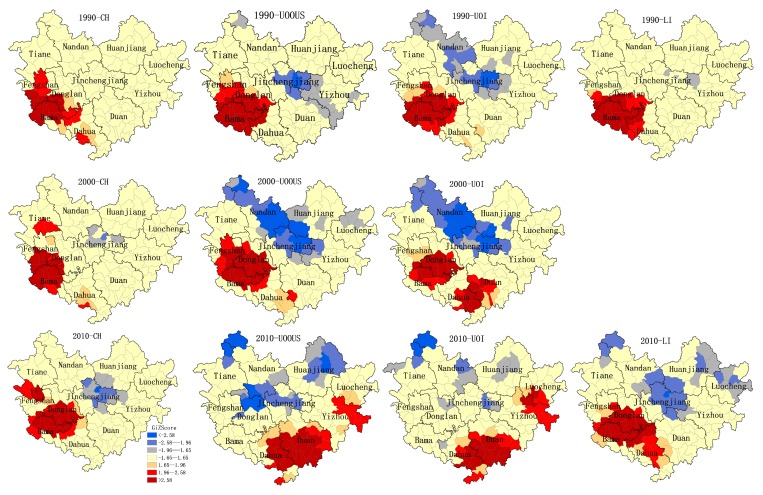
Hot and cold clusters of CH, UOOUS, UOI and longevity index (LI) using Getis-Ord G* statistics.

Results of the statistical analysis in Figure 3 and Table 1 indicate that different indicators and times show different spatial aggregation characteristics. Overall, the results in Table 1 suggest that the distribution of longevity indicators clustered in all four demographics. The global Moran’s *I* from 1990 to 2010 showed a distinctive and significantly positive spatial autocorrelation. In 1990, the index difference of Moran’s *I* can be expressed as follows: UOI > UOOUS > LI > CH. A more significantly positive spatial autocorrelation of UOI and UOOUS indicated a better aggregation state of high-value and low-value, compared with LI and CH. This result indicated that the clustering of the extremely long-living population was not prominent during this period.

A rapid increase in the clustering of UOOUS was observed in 2000, while CH retained a weaker spatial autocorrelation. Moreover, the longevity of the elderly population has a strong spatial dependence. This phenomenon is even more pronounced in 2010 as LI > UOOUS > CH > UOI. The longevity ratio based on the whole population is relatively stochastic, although these factors still exhibit a significant spatial autocorrelation. From the perspective of vertical time, Moran’s *I* of UOI and UOOUS show a consistent pattern of change (2000 > 1990 > 2010) (see Table 1), whereas, the variation in CH and LI shows the contrary (2010 > 1990 > 2000). Such outcomes suggest that the spatial accumulation of the extreme longevity population is increasing.

We used Getis-Ord G* to identify the hotspots of longevity incidence. If Gi* > 1.96 or 2.58 (as is shown in the red part of Figure 3), the longevity incidence of the region is statistically significant (*p* < 0.05 or *p* < 0.01) and the region is regarded as a high-incidence area (namely, a hotspot). The hotspots of CH are evidently concentrated in the southwestern part of Hechi (see Figure 3). Over time, the hotspots continued to expand and continuously spread to Donglan County. Nine stable hotspots of CH were identified in data from three population censuses (Figure 3). The hotspots of LI also showed similar patterns. The two population censuses in Donglan County indicated that its hotspots are increasing, while Bama exhibited the opposite result. Figure 3 illustrates that UOI and UOOUS display almost the same hotspot patterns. The hotspots were initially concentrated southwest of Hechi and subsequently spread to the southeast. By 2010, the hotspots completely transferred to the eastern and southern parts of Hechi. This change is basically consistent with the results in Figure 2. Large numbers of overlapping areas were detected in the hotspots of UOI and UOOUS in 2010, which are the new hotspots of concern.

### 3.3. Gravity Center Migration of the Longevity Phenomenon

**Figure 4:**
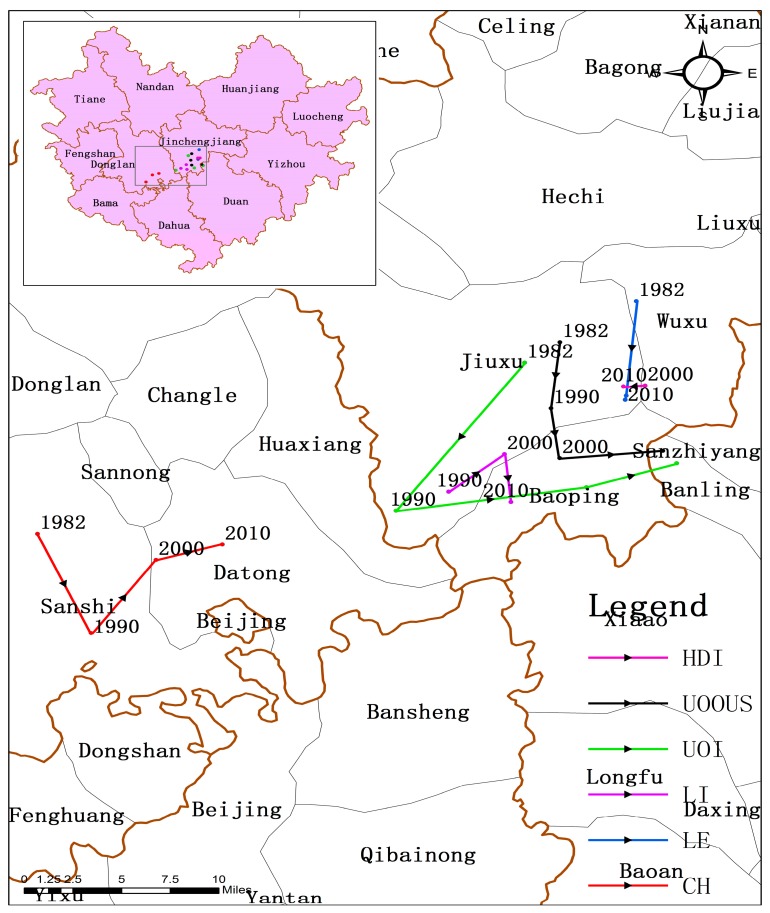
The curve of gravity center migration on longevity indicators (CH, UOOUS, UOI and LI) and HDI.

Gravity modeling indicates that, from 1982 to 2010, Donglan County was the gravity center of CH and the center continues to migrate eastward (Figure 4). This result suggests that centenarians are mainly concentrated in the southeastern part of Hechi. The movement curves of the gravity center in terms of UOI and UOOUS are similar, initially moving to the southwest (1982–1990) and subsequently progressing to the east (1990–2010).

Such a finding indicates that the gravity center of the population ratio over 80 years of age moved first to the southwest, followed by long-term migration to the east. The result is verified in Figure 2 and Figure 3. In general, the gravity center of LI moves eastward, whereas the north-south movement is not obvious. Notably, the direct movement trajectory of LI is at the gravity center of HDI and infinitely close to it. This outcome may be related to the affinity between the two factors. Overall, the location of HDI’s gravity center changes slightly, whereas the other indicators move closer to it (Figure 4). In addition, the gravity centers do not match the geometric and government centers. This finding also supports the previous results that the distribution of longevity is not random.

### 3.4. Simple Linear Correlation between the Research Indicators

The above results show that life indicators have tendency to shift to the east and south of Hechi. At the same time, LE’s high-value areas and the gravity center of HDI are distributed in the mid-eastern regions of Hechi. Thus, we calculated the correlation coefficients between longevity indicators (CH, UOI, UOOUS, and LI) and LE, as well as lifespan indicators (CH, UOI, UOOUS, LI, and LE) and HDI. The Pearson’s *r* among the indicators in 2010 are: CH and HDI (−0.645), LI and HDI (−0.723), UOI and HDI (−0.844). A significantly negative correlation between the indicators was identified. However, a correlation between longevity indicators and LE was not confirmed. A slightly significant correlation was detected in UOOUS, LE (2010), and HDI. However, the correlation between LE and HDI was completely positive in 2000 unlike its correlation in 2010 (see Table 2).

## 4. Discussion

China, a populous country with a complex geographical environment, diverse climate, and unique economic growth model, is also a valuable academic subject in the context of the population problem. In the past few decades, several longevous areas have been identified in China, such as Zhongxiang (Hubei Province), Xiayi (Henan Province), Bama (Guangxi Province), and Leshan (Sichuan Province) [38]. Among these areas, Bama has been studied extensively in recent years as a famous longevous area [10,39]. Previous studies showed that the unique geographical environment of the area and genetics are vital to the longevity of the population in Bama. Based on the analysis of the local food and drinking water, four characteristic elements are closely related to the health of elderly people from Bama, including Cr, Fe, Mn and Co [40]. The specific metabolic pattern of centenarians may crucially and positively influence the formation of the longevity phenomenon [41,42]. Elevated dietary fiber intake should be a path toward health and longevity [43]. Furthermore, the family aggregation phenomenon also affects the longevity of Bama. Several centenarians at the same period belong to a large family, suggesting that longevity is closely related to genetics. This conjecture was validated in series of subsequent studies [11,44,45].

Results of this study revealed that other longevous areas besides Bama exist in the Hechi district. In the last four censuses, a stable longevous pool was observed in the southwestern part of Hechi, including Bama, Donglan and Fengshan (Figure 2 and Figure 3). Over time, the range of longevity expands, followed by the increase of CH and LI. The three townships may exhibit homogeneous longevity patterns because of their similar geographical environments, the karst landscape, and the red water basin. It is more likely that this is an artefact caused by the arbitrary borders of the different regions. This finding suggests that there may be stable factors in this region that promote longevity. From the geographical point of view, the longevity hotspots belong to a mountainous region, which was quite remote and difficult to access until several mountain roads were built a few decades ago. This geographic situation discouraged immigration, favored inbreeding, intermarriage, and consanguinity, thus decreasing the variability of the genetic pool. The unique geographical environment and cultural characteristics of this zone (including dietary habits and lifestyles) can be considered as shared characteristics favoring extreme longevity. Additionally, this pattern is similar with that observed on the island of Sardinia (Italy) [46,47].

UOI and UOOUS represent the proportion of the elderly over 80 years of age in different groups. Evident spatial clustering and variation in the two indicators were observed. The high-value longevity area gradually shifted from the southwest to the south and east and formed a new longevous area. This change is likely related to regional imbalances in socio-economic development. A positive socio-economic development state often implies improved living conditions, increased access to health services, more job opportunities, and higher income, which, in turn, reduces mortality and significantly improves human lifespan [48,49]. This condition has led to a significant augmentation in the proportion of centenarians and elderly people over the age of 90 and 80 during the past three decades. At the same time, the life expectancy of the population in Hechi City also gradually increased in line with other longevity indicators. The LE in the northeastern region is always higher than that in other regions in the same period, which may be attributed to the economic development brought about by the increase in industry in Yizhou, Luocheng. Meanwhile, the life expectancy of Bama and Fengshan increased rapidly in the fourth census (2010). We surmise that this phenomenon may be related to the development of the tourism industry in the past decade. Large numbers of longevity-based commodity exports provide additional economic income to the people of this area, although these residents still retain the original farming economy and cultural customs, such as diet, daily routines, and optimism [50,51]. The influence of socio-economic development on longevity can also be observed from the above migration of the gravity center. Almost all the life indicators migrate to the southeast and continue to gather in terms of HDI. The gravity center of HDI is highly consistent with that of LE. This finding suggests that socio-economic development has a certain role in promoting human health and longevity. From a clustering variation point of view, the proportion of the elderly over 80 years of age shows a significant increase in the eastern and southern areas, whereas the proportion of those aged 90 years and above and of centenarians did not change significantly in the above areas. This result may be explained by the Preston curve model [52], which indicates that further increase of socio-economic development was associated with relatively large gains in the lifespan at low levels of socio-economic conditions, whereas the increased level of socio-economic development had little relation with the variation of the lifespan at high socio-economic levels. In other words, while socio-economic development cannot infinitely increase human life, it can help people achieve a relatively advanced age; however, its influence on exceptional longevity is limited.

A significant statistical relationship was not observed between longevity indicators and life expectancy. Meanwhile, the longevity index was significantly and negatively correlated with socio-economic development in 2010. Similar result was obtained by Wang [38,53]. Socio-economic development plays a role in promoting life, but this function cannot be proven statistically. The small sample size of this study may be one of the possible reasons. In addition, we must consider the negative effects of socio-economic development, as well as its benefits, because of the diversity of longevity factors [54]. Socio-economic development may result in environmental pollution, unreasonable distribution of resources, unemployment, and other problems, all of which are potentially harmful to health and may result in irrelevance of life indicators to economic development [55,56]. Hechi is famous for its rich reserves of non-ferrous metals, and almost all the mining industries are concentrated in Northern Nandan, Tiane, and Luocheng. Although industrial development led to the improvement of the local economy, it may also undermine the natural environment, thus adversely affecting people’s health. There is a perfect correlation in 2000, and no correlation in 2010. This result may be attributed to overall unbalanced development in all aspects of the county, such as economics, gender, base facilities and education. The imbalances of the access to social support and medical can also lead to inequality of health and longevity [57,58]. We speculate that the negative effects of development may explain why the socio-economic level increase in these areas while the level of longevity shows the opposite outcome. Further research is necessary to confirm this inference.

There are several shortcomings in our research. Firstly, the quality of the census, especially the authenticity of the age, is of concern to many scholars. Although studies have shown that the quality of censuses in China over the past three decades is high, the verification of the age of ethnic minorities involved in this study is rarely reported [59,60]. This is exactly what we plan to do next. Second, due to the availability of data, the sample size of the correlation analysis in this study is small and the selected research factors are few, which may lead to a certain degree of bias. Last but most important, our study is based on county-specific or even township-specific data, which are small area data, barely relying on them will bring some instability to the results [61].

## 5. Conclusions

Lifespan indicators (CH, LI, UOI, UOOUS, and LE) can describe the spatial distribution and reflect the variation patterns of longevity from multiple dimensions. The geographical scope of longevity in Hechi City is gradually expanding, and significant spatial clustering was observed in the southwestern, southern, and eastern parts of the city. This study also found that social economic development is likely to have a certain impact on new longevous areas, but the role of which in extreme longevity is not significant.

## Figures and Tables

**Table 1:** Global spatial autocorrelations of lifespan indicators from 1982 to 2010.

Longevity Indicators	Year	Moran’s *I*	*Z*	*p*-Value
CH	1990	0.404	7.896	<0.0001
	2000	0.403	7.927	<0.0001
	2010	0.505	9.403	<0.0001
UOI	1990	0.638	11.599	<0.0001
	2000	0.669	12.229	<0.0001
	2010	0.457	8.418	<0.0001
UOOUS	1990	0.627	11.475	<0.0001
	2000	0.729	13.395	<0.0001
	2010	0.554	10.120	<0.0001
LI	1990	0.516	9.740	<0.0001
	2010	0.681	12.604	<0.0001

CH: number of centenarians per one hundred thousand inhabitants; UOI: ultra-octogenarian index; UOOUS: ultra-octogenarian of the ultra-sexagenarian; LI: longevity index.

**Table 2:** Pearson correlation coefficients between longevity indicators and LE, as well as lifespan and Human Development Index (HDI).

Lifespan Indicators	Year	Pearson *r*	*p*-Value	*N* *
CH and LE	1982	−0.612 ^a^	0.060 ^a^	10
	2000	−0.156	0.648	11
	2010	−0.002	0.995	11
LI and LE	2000	−0.135	0.692	11
	2010	0.050	0.884	11
UOI and LE	1982	−0.131	0.718	10
	2000	−0.175	0.607	11
	2010	0.198	0.560	11
UOOUS and LE	1982	−0.066	0.857	10
	2000	−0.211	0.534	11
	2010	0.461	0.154	11
CH and HDI	2000	−0.156	0.648	11
	2010	−0.645	0.032 **	11
LI and HDI	2000	−0.135	0.692	11
	2010	−0.723	0.012 **	11
UOI and HDI	2000	−0.175	0.607	11
	2010	−0.844	0.001 **	11
UOOUS and HDI	2000	−0.211	0.534	11
	2010	−0.451	0.164	11
LE and HDI	2000	1.000	0.000 **	11
	2010	0.090	0.793	11

^a^ Spearman’s rank correlation; * Numbers of county-level administrative units; ** *p* < 0.05. LE: life expectancy at birth.

## Supplementary Materials

The following are available online at www.mdpi.com/1660-4601/14/7/812/s1, Figure S1: Spatial and temporal distribution of CH, LI, UOI and LE in Hechi city at county level (1982–2010).
