# Supplementary figures and images for: Spatio-Temporal Variation of Longevity Clusters and the Influence of Social Development Level on Lifespan in a Chinese Longevous Area (1982–2010)
Source: Int J Environ Res Public Health. 2017 Jul 19;14(7):812. doi: 10.3390/ijerph14070812 (PMC5551250; doi:10.3390/ijerph14070812)

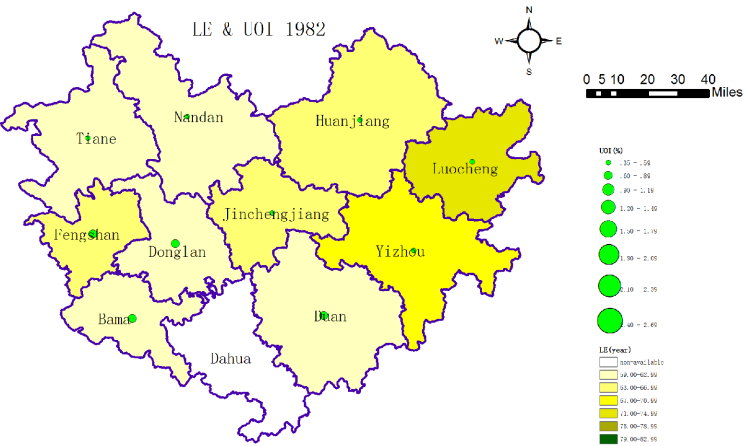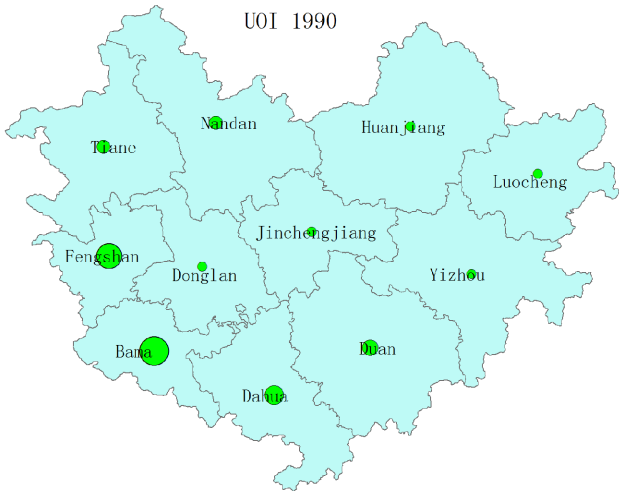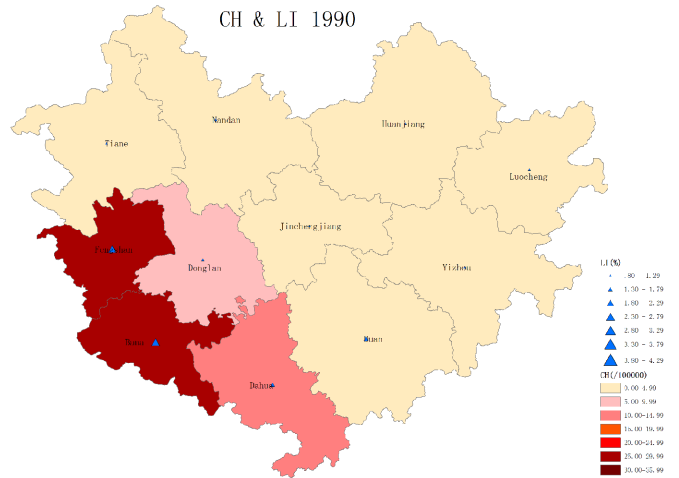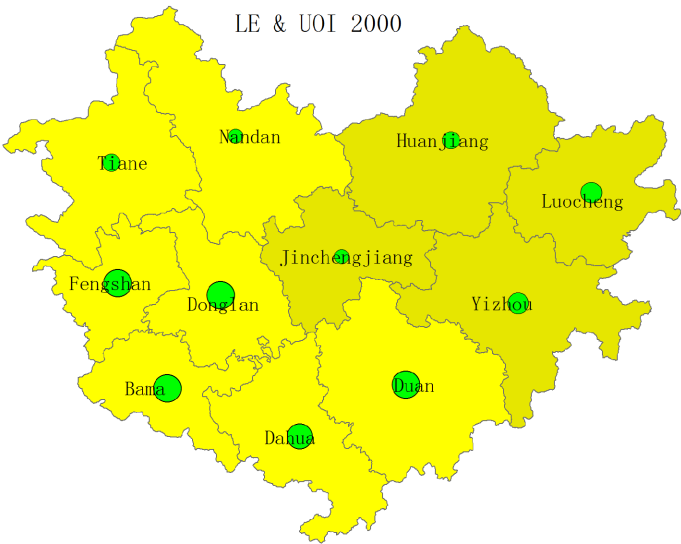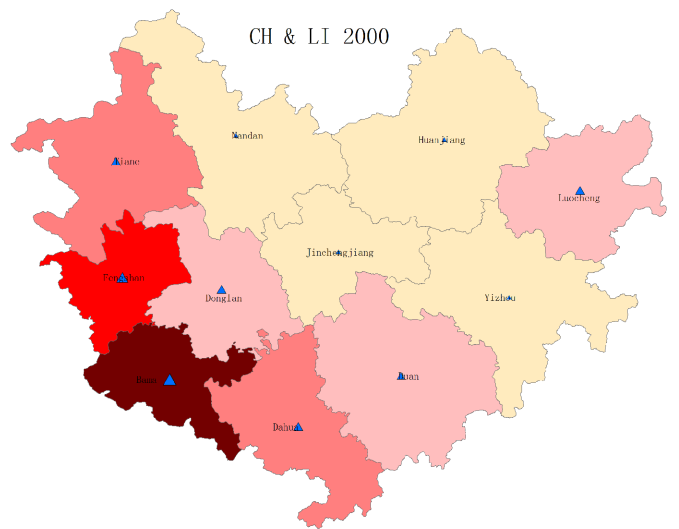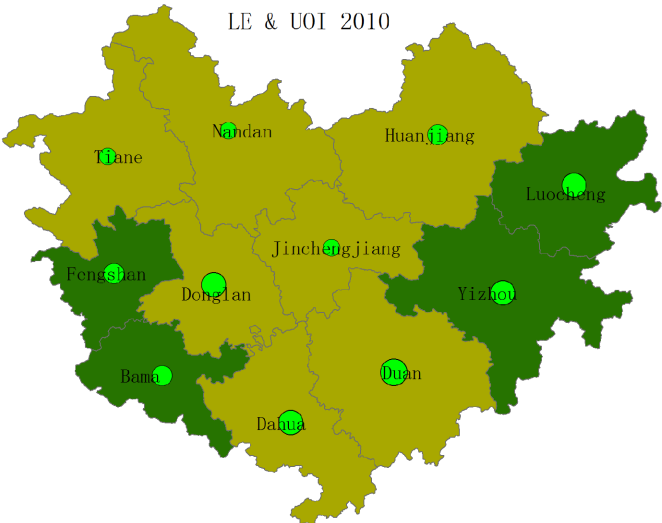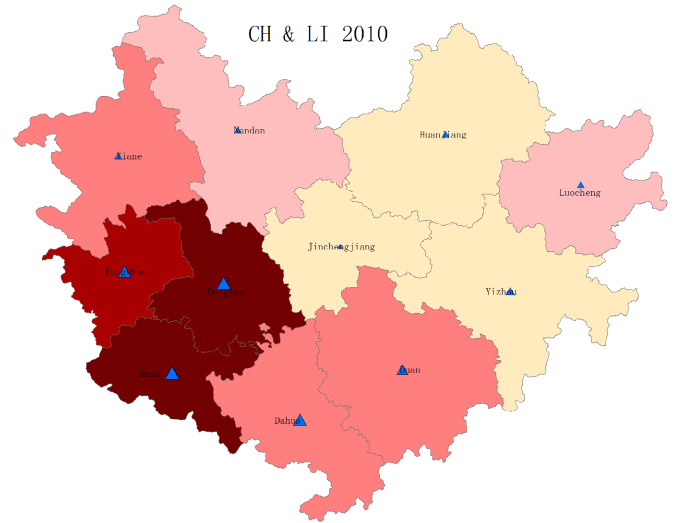

Supplement: Supplementary file 1 [file ijerph-14-00812-s001.pdf]
